# Blockage of S100A8/A9 ameliorates septic nephropathy in mice

**DOI:** 10.3389/fphar.2023.1172356

**Published:** 2023-07-20

**Authors:** Wei Shi, Tian-Tian Wan, Hui-Hua Li, Shu-Bin Guo

**Affiliations:** Beijing Key Laboratory of Cardiopulmonary Cerebral Resuscitation, Department of Emergency Medicine, Beijing Chaoyang Hospital, Capital Medical University, Beijing, China

**Keywords:** acute kidney injury, Paquinimod, mitochondrial dynamics, S100A8/A9, sepsis

## Abstract

Septic acute kidney injury (AKI) is the commonest cause of complication of sepsis in intensive care units, but its pathophysiology remains unclear. Calprotectin (S100A8/A9), which is a damage-associated molecular patterns (DAMPs) molecule, exerts a critical role in modulating leukocyte recruitment and inflammatory response during various diseases. However, role of S100A8/A9 in septic AKI is largely unknown. In this research, Septic AKI was triggered by cecal ligation and puncture (CLP) operation in wild-type mice, which treated with or without an S100A9 inhibitor, Paquinimod (Paq, 10 mg/kg) that prevents S100A8/A9 to bind to Toll-like receptor 4 (TLR4). Renal function, pathological changes, cell death, and oxidative stress were evaluated. Our research indicated that the mRNA and protein expression of S100A9 are time-dependently elevated in the kidney following CLP. Moreover, the administration of Paq for 24 h significantly improved CLP-induced renal dysfunction and pathological alterations compared with vehicle treatment in mice. These beneficial effects were associated with the inhibition of CLP-triggered renal tubular epithelial cell apoptosis, inflammation, superoxide production, and mitochondrial dynamic imbalance. What’s more, we further confirmed the above findings by cell co-culture experiments. Our study demonstrates that S100A9 is a prominent protein to lead to septic AKI, and the selective inhibition of S100A9 could represent a new therapeutic approach which can treat septic AKI.

## 1 Introduction

Sepsis is a systemic inflammatory response in which the host is deregulated by infection. Sepsis is a common disease with a high mortality rate, and acute kidney injury (AKI) is the leading complication in intensive care units, accounting for 45%–70% of all AKIs (Sun et al., 2019). Increasing evidence suggests that multiple pathophysiological processes are involved in the development of septic AKI, including hypoperfusion-induced ischemic injury, microvascular dysfunction, intrarenal redistribution of renal blood flow in the kidney, infiltration, and activation of immune cells, massive release of inflammatory cytokines, and endocrine dysregulation (Quaglia et al., 2022). Although the molecular mechanism of septic AKI is complex, microvascular impairment can alter the function and density of capillaries, leading to intrarenal shunts and renal ischemia. Moreover, inflammation takes a vital part in producing excessive oxidative stress, apoptosis, as well as mitochondrial dysfunction in renal tubular epithelial cells (So et al., 2022). Therefore, it is crucial to identify the key signaling pathways involved in inflammation and mitochondrial dysfunction during septic AKI.

The S100 protein family is an effective amplifying factor for inflammatory responses. S100A8 and S100A9, which belong to the S100 family, are cytoplasmic EF-hand Ca^2+^-binding proteins. They form a heterodimer that is highly expressed in activated neutrophils and monocytes/macrophages. S100A8/A9 protein is widely distributed in human cells, tissues, and body fluids and can be detected in serum, urine, and cerebrospinal fluid (Tammaro et al., 2018; Wang et al., 2018). S100A9 consists of 110 amino acids with a molecular weight of ∼13 KDa (Wang et al., 2018). S100A8/A9 is the most abundant damage-associated molecular patterns (DAMPs) molecule [14]. It can tie to toll-like receptor 4 (TLR4) or the receptor for advanced glycation end products (RAGE) to activate JAK/STAT, PI3K/AKT, MAPK/NF-κB, and NLRP3 inflammasome pathways, thereby enhancing proinflammatory response and development of various inflammatory diseases, including autoimmune disease, chronic obstructive pulmonary disease, and cardiovascular disease (Wang et al., 2018). Moreover, studies have reported that S100A8/A9 is related to the great variety of kidney diseases, including ischemia/reperfusion (I/R)-induced AKI, contrast-induced AKI, obstructive and diabetic renal fibrosis, acute urinary tract infection, and glomerulonephritis in different animal models (Dessing et al., 2010; Pepper et al., 2015; Tan et al., 2017; Tammaro et al., 2018; Du et al., 2022; Yao et al., 2022). Further, emerging bioinformatics studies point that S100A9 may act as a potential biomarker and therapeutic aim for septic shock-related renal injury (Tang et al., 2021). While, the role and mechanism of S100A8/A9 in the genesis of AKI in sepsis is poorly understood.

In this research, using Paquinimod (Talley et al., 2021; Zhao et al., 2021; Su et al., 2022) (Paq, also known as ABR25757), an S100A8/A9 specific inhibitor which prevents S100A8/A9 to bind to TLR4, we explored the role of S100A9 in the development of septic AKI in a murine model of cecal ligation and puncture (CLP)-induced sepsis. We found that S100A9 level was notably regulated upwards in the kidney of CLP-treated mice during 24–72 h. CLP-induced renal dysfunction and pathological alterations, including tubular epithelial cell apoptosis, inflammation, and oxidative stress, was ameliorated by using Paq to inhibition of S100A9 in mice. These preventive effects were associated with the inhibition of mitochondrial fission-fusion imbalance by the reduction of dynamin-related protein 1 (Drp1) and an increase in mitofusin-1/2 (Mfn1/2). In addition, we further confirmed the above findings by cell co-culture experiments. Therefore, our results suggest that S100A8/A9 likely has an effect on CLP-induced AKI by impairing Drp1/Mfn1/2-mediated mitochondrial balance, and reducing and suppressing S100A9 may represent a new therapeutic method for septic AKI.

## 2 Methods and materials

### 2.1 Animals

C57BL/6J wild-type (WT) mice were bought from Charles River (China). All mice were cultured in Medical Research Center at the Beijing Chaoyang Hospital (China). They are fed in specific pathogen-free facility at temperature about 25°C–26°C with the condition of 12-hour light-dark cycle. Only male mice were recruited in this paper. All animal experimental procedures are endorsed by the Animal Care and Use Committee of Chao-Yang Hospital (2021-Animal-35) and complied with regulations for the Care and Use of Laboratory Animals prepared by the U.S. *NIH.*

### 2.2 Cecal ligation and puncture (CLP) operation

Male mice (8 weeks, 22–24 g) were fed for 1 week to facilitate adaptation to their surroundings, and a murine model of sepsis was made by CLP operation as described [20]. Then, the mice (*n* = 6 per group) were treated with 2.5% tribromoethanol (0.01 mL/g; Sigma) via intraperitoneal injection. Further, mid-abdominal laparotomy was performed to expose the abdominal organs, and the cecum was ligated at 1/2 and punctured with a 21-gauge needle. Control-group animals did not undergo ligation and puncture.

### 2.3 Paquinimod treatment

Paquinimod (Paq, HY-100442, MCE, NJ), an S100A9-specific small molecule inhibitor that prevents S100A8/A9 to bind to TLR4, was dissolved directly in castor oil (vehicle) and administered to the mice at dosages of 10 mg/kg by intraperitoneal injection 1 day before and after CLP surgery. The optimal dose of Paq (10 mg/kg) was determined based on a literature review as described (Talley et al., 2021) and our preliminary data. To measure the content of S100A9 mRNA and proteins in the kidney, mice were randomly assigned into four groups and was performed with CLP surgery for 24–72 h (sham, CLP-24 h, CLP-48 h, CLP-72 h, *n* = 6 per group). To examine the role of Paq in our experiment, the mice were subjected to CLP surgery for 48 h (sham + vehicle, sham + Paq(10 mg/kg),CLP + vehicle, CLP + Paq (10 mg/kg), *n* = 6 per group). The sham mice were given an injection intraperitoneally with the same volume of castor oil (vehicle) alone (*n* = 6 per group). The kidney and blood samples were collected to examine their histology, the markers for renal function, and the gene mRNA and protein levels.

### 2.4 Cell co-culture *in vitro*

The cell lines of mouse monocyte/macrophage cell line (RAW264.7; BNCC354753) and mouse renal tubular epithelial cells (mRTECs; BNCC3660478) were purchase from BeNa Culture Collection (BNCC, Beijing). They were cultured in cell incubators at 37°C, 5% CO_2_, and 95% air. RAW264.7 cells were inoculated in the upper chamber and mRTEC cells were seed in lower chamber of 24-well transwell cell culture (cell number about 1×10^6^). After cell adherence, RAW264.7 were pretreated with LPS (50 μg/mL) for 24 h. The lower chamber containing mRTEC were pretreated with Paq (150 μM) for 2 h before co-culture of the 2 cells.

For the examination of S100A9 from RAW264.7 cells on mitochondrial fusion and fission related proteins in mRTECs (*n* = 3), 2 cell lines were co-cultureed for 24 and the mRTEC cells were examined using double immunostaining with anti-Drp1 (CST, China, 1:50), anti-Mfn-1 (Proteintech, United States, 1:100), anti-Mfn-2 (Proteintech, 1:100), and anti-ATP5A (Proteintech, United States, 1:300). All images were randomly selected from 5 visual fields for each slice and photographed with a Nikon microscope (Nikon, Tokyo).

For the examination of S100A9 from RAW264.7 cells on oxidative stress and apoptosis, the mRTEC cells (*n* = 3) were stained with dihydroethidine (DHE, Sigma), anti-cleaved caspase-3 (CST, China, 1:400), respectively. Meanwhile, the RAW264.7 cells were treated with LPS in different concentrations (0,1, 10, 50 μg/mL) for 24 h, and supernatants were absorbed (1,000 rpm, 20 min) to analyze the contents of S100A9, IL-6 or TNF-α with ELISA kits.

### 2.5 Immunofluorescence double staining analysis

For the double immunostaining of kidney tissue, anti-S100A9 (Abcam, United States, 1:1000) and anti-F4/80 (Abacam, 1:400) were used to stain kidney tissue section at 48 h of CLP surgery.

### 2.6 Renal function measurement

Blood samples (*n* = 6 per group) were obtained from the eyeballs of the mice in each group, stratified, and centrifuged (3,000 rpm, 15 min). Aspirate the upper supernatant and save in a centrifuge tube. The levels of renal function markers were determined using BUN and Cr assay kits (Nanjing Jian cheng) [20].

### 2.7 Histology analysis

Kidney tissues (*n* = 6 per group) were infiltrated in 4% paraformaldehyde, fixed for 24 h, inserted in paraffin, cut into 5 μm thick slices, and placed on glass slides for hematoxylin and eosin (H&E) and periodic acid-Schiff (PAS) staining as described previously (Wei et al., 2019). All images were randomly selected from 15 visual fields for each slice and photographed. The slices for acute tubular necrosis (ATN) were blindly scored to evaluate the pathological changes, which accounted for the total number of renal tubules.

### 2.8 Immunohistochemistry analysis

The expression levels of S100A9,F4/80, ki67 on the renal sections were determined using immunohistochemistry as previously described (Chen et al., 2022). These expression levels were determined with anti-S100A9 (Abcam, United Kingdom, 1:500), anti-F4/80 antibody (Abcam, United Kingdom, 1:100) or anti-ki67 (Abcam, United Kingdom, 1:300). After the slices were dewaxed, they were incubated with anti-S100A9, anti-F4/80 antibody or anti-ki67 overnight at 4°C. Then, the color reaction was performed using a diaminobenzidine (DAB) coloring kit (Gene Tech, Shanghai, China). All images were randomly selected from 5 visual fields for each slice and photographed with a Nikon microscope (Nikon, Tokyo).

### 2.9 Analysis of apoptosis and oxidative stress

For detection of cell apoptosis, the frozen kidney sections (*n* = 6 per group) were processed with situ Cell Death Detection Kit (Roche Group) and diamidino-2-phenylindole (DAPI, Sigma) according to the protocols from manufacturer. For superoxide examination, the frozen kidney sections (*n* = 5 per group) were processed with 1 μmol/L dihydroethidine (DHE, Sigma) in phosphate-buffer saline buffer for 30 min based on the protocols from producer. H_2_DCFDA is also an indicator of oxidative stress. Ice slices were made from fresh kidney tissue without settling sugar and stained with H_2_DCFDA for 5 min (*n* = 5 per group), washes three times in PBS. Fluorescence images were randomly selected from 5 visual fields and photographed. The percentage of TUNEL+ nuclei (red) to DAPI-stained nuclei (blue) was calculated.

### 2.10 Quantitative real-time PCR analysis

Total RNA was acquired from the fresh kidney of sham- or CLP-operated mice with chloroform, isopropyl alcohol, and TRIzol reagent (Takara, Japan) based on the protocols from the manufacturer. A reverse transcription kit (Takara) was applied to compound cDNA. Remove genome reaction and reverse transcription reaction conditions were set at 42°C for 2 min and 37°C for 15 min, respectively. The relative mRNA content of IL-6, TNF-α, NOX1, NOX2, and GAPDH were detected. The mRNA levels were standardized to the GAPDH level and statistically analyzed using the 2^−ΔΔCT^. All the primer sequences were acquired from Sheng gong Biotech Co., Ltd. (Shanghai, China) and are presented in Table 1.

**TABLE 1:** Primer sequences for q PCR analysis.

Gene	Gene ID	Sequences
S100A9	20,202	5′-ATA​CTC​TAG​GAA​GGA​AGG​ACA​CC-3′
		5′-TCC​ATG​ATG​TCA​TTT​ATG​AGG​GC-3′
IL-6	16,193	5′-GCT​ACC​AAA​CTG​GAT​ATA​ATC​AGG​A-3′
		5′-CCA​GGT​AGC​TAT​GGT​ACT​CCA​GAA-3′
INF-α	21,926	5′-ATG​GCC​TCC​CTC​TCA​TCA​GT-3′
		5′-CTT​GGT​GGT​TTG​CTA​CGA​CG-3′
NOX-1	237,038	5′-AGC​CAT​TGG​ATC​ACA​ACC​TC-3′
		5′-AGA​AGC​GAG​AGA​TCC​ATC​CA-3′
NOX-2	13,058	5′-TGC​ACC​ATG​ATG​AGG​AGA​AA-3′
		5′-CCA​CAC​AGG​AAA​ACG​CCT​AT-3′
GAPDH	14,433	5′--CAT​CAC​TGC​CAC​CCA​GAA​GAC​TG-3′
		5′-ATG​CCA​GTG​AGC​TTC​CCG​TTC​AG-3′

### 2.11 Western blot analysis

One-fifth of the kidney tissues were placed in a mixture of 200 μL RIPA (Beyotime, Shanghai) and 2 μL cocktail (Thermo Fisher), minced, and homogenized on ice. Lysates were then centrifuged at 13,300 g for 15 min at 4°C. The clarified supernatant was saved and subsequently analysed for content by using a Bicinchoninic Acid (BCA) assay kit (Thermo Fisher, United States). Equal amounts (40–50 μg) of the supernatants were split in 10% and 15% SDS-PAGE, transferred to membranes, and incubated with primary antibodies overnight at 4°C. The optical density was detected with enhanced chemiluminescence (ECL) reagents (Bio-Rad Laboratories) using a Fluorchem R device, and the protein content were standardised to the GAPDH level. The primary antibodies used for Western blotting were as follows: S100A9 (Abcam, United Kingdom, 1:500), Drp1 (CST, American, 1:2000), Mfn1 (CST, American, 1:2000), Mfn2 (CST, American, 1:2000), Bax (Proteintech, American, 1:5000), Bcl-2(Proteintech, American, 1:1000) and β-actin (CST, American, 1:1000).

### 2.12 Measurement of caspase-3 activity

Total 50 mg of kidney from sham-or CLP-operated mice were placed in the liquid provided with the kit and homogenized on ice. The protein content was measure with Bradford method (Beyotime Biotechnology, Shanghai, China). Caspase-3 activity levels were acquired by caspase-3 assay kit (Solarbio, Beijing, China) based on the formation of the chromophore p-nitroaniline (p-NA) by cleavage from the labeled substrate DEVD-pNA following the protocols from the manufacturer. This analysis was used to further confirm the data of immunostaining with cleaved caspase-3 antibody, and TUNEL kit.

### 2.13 Measurement of S100A9,IL-6,TNF-α and malondialdehyde with ELISA kits

Total 50 mg of kidney from sham- or CLP-operated mice (*n* = 5 per group) were placed in normal saline and homogenized on ice. The homogenate was absorbed (3,000 rpm, 10 min, 4°C) to further analyze. The clarified supernatants were dilute 50 times. Content of protein was measure using a Bicinchoninic Acid (Thermo Fisher, United States). The levels of malondialdehyde (MDA) were determined with MDA assay kits (Nanjing Jiancheng). Total 40 mg of kidney tissue from sham- or CLP-operated mice (*n* = 5 per group) were placed in fresh lysis buffer, homogenized on ice and then were centrifuged at 10,000 rpm for 5 min to detect the content of IL-6 and TNF-α(Cloud-Clone Crop, Wuhan, China). Blood samples (*n* = 5 per group) were obtained from the eyeballs of the mice in each group, stratified, and centrifuged (3,000 rpm, 15 min). Supernatant were aspirated and saved in a centrifuge tube to measure the level of S100A9 (Cloud-Clone Crop, Wuhan, China).

### 2.14 Statistical analysis

Statistical analysis was processed by using the SPSS 20 software, and the results are presented as the mean ± SEM. Statistical differences between the two groups were analysed by using an unpaired two-tailed Student’s t-test. Comparisons between multiple groups were analysed by using a one-way ANOVA and subsequently a *post hoc* test. Survival rates in each group were analyzed with the Gehan-Breslow and log-rank tests (*n* = 39–59 animals per group). *p*-value of <0.05 is considered with significant.

## 3 Results

### 3.1 S100A9 is regulated upwards in the kidneys of the CLP-operated mice

**FIGURE 1:**
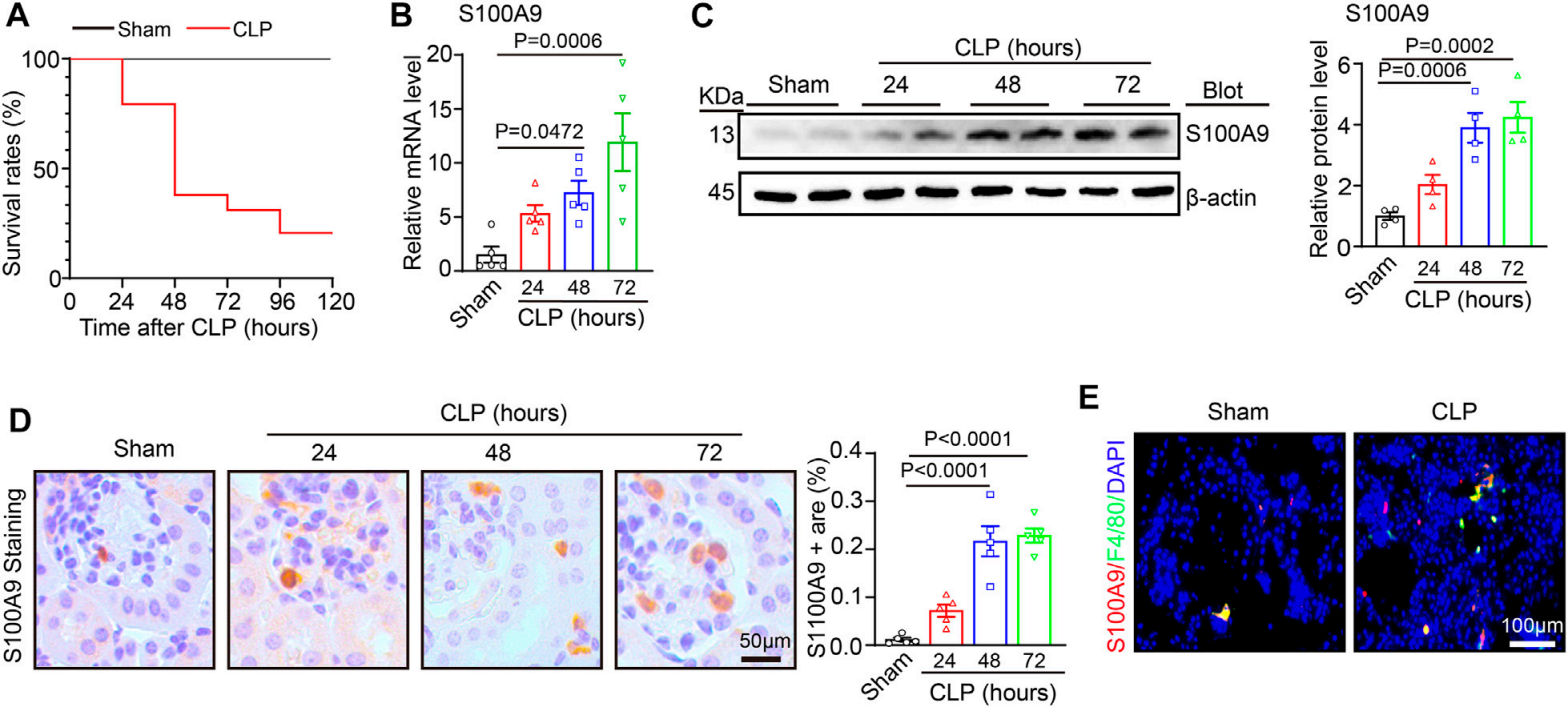
S100A9 expression level in the kidneys of the mice after cecal ligation and puncture (CLP) surgery: **(A)** male wild-type (WT) mice were applied to sham or CLP operation for 24–120 h. The survival rates of each group at different time points are shown (Sham, *n* = 5; CLP, *n* = 29). **(B)** Quantitative polymerase chain reaction analysis of S100A9 mRNA content in the kidneys of the mice at different time points (*n* = 5). **(C)** Immunoblotting analysis of S100A9 in the kidneys of the mice at different time points (left) and quantifying the protein level (*n* = 4). **(D)** Immunohistochemical staining of S100A9 in the kidneys of the mice at different time points (*n* = 5). **(E)** Immunofluorescence double staining of kidney tissue with S100A9 and F4/80 antibodies at 48 h of CLP surgery. Scale bar: 50–100 μm.

To investigate the effect of S100A9 in septic AKI, we generated a sepsis-associated AKI model by CLP surgery. Then, we evaluated the survival condition of the mice at 24, 48, 72, 96, and 120 h after the surgery. Compared with sham controls, the survival rates of the CLP-treated mice were 100%, 80%, 38%, 31%, and 21%, respectively (Figure 1A). Then, the kidney specimens were collected at 24, 48, and 72 h after the surgery, and S100A9 expression was assessed. qPCR and immunoblotting analysis indicated that S100A9 content at both mRNA and protein was time-dependently regulated upwards in the kidneys of CLP-treated mice after 24, 48, and 72 h (Figures 1B, C). The increased expression of S100A9 proteins in the kidneys of CLP-treated mice was further demonstrated via immunohistochemical staining with an anti-S100A9 antibody. Most S100A9 proteins were distributed in the glomerulus and tubulointerstitium (Figure 1D). Moreover, previous studies suggest that S100A8/A9 was colocalized with Ly6G-positive granulocytes in ischemia/reperfusion (I/R) kidney and obstructive nephropathy (Dessing et al., 2015; Tammaro et al., 2018). Our immunostaining further demonstrated that S100A9 was also colocalized with F4/80-positive macrophages in CLP-treated renal tissue (Figure 1E). Thus, these findings suggest that the upregulation of S100A9 may exert a role in septic AKI.

### 3.2 Administration of Paquinimod improves CLP renal dysfunction in mice

**FIGURE 2:**
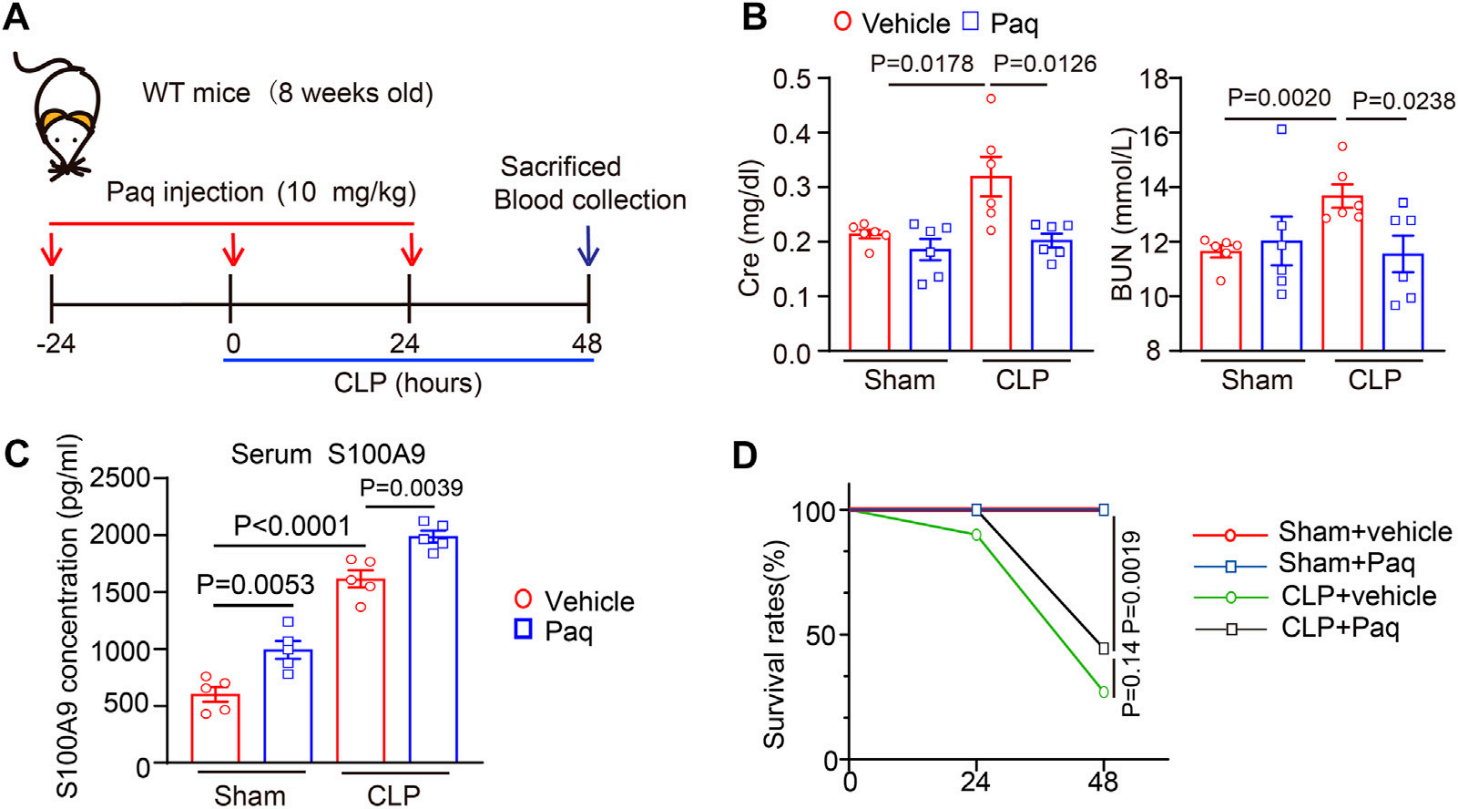
Administration of Paq to mice improves CLP-induced renal dysfunction. **(A)** Schematic representation for the administration of Paq and CLP surgery. **(B)** The WT mice were pretreated with Paq at dosages of 10 mg/kg at 24 h before CLP surgery and after 48 h. The content of serum urea nitrogen (BUN) and creatinine (Cr) were tested by using the ELISA kit (*n* = 6 per group) **(C)** The level of serum S100A9 were tested by using the ELISA kit (*n* = 5 per group). **(D)** Survival rates of vehicle- or Paq- (*n* = 10, 10 mg/kg) treated mice after sham and CLP surgery (*n* = 9–10 per group).

To determine the role of S100A9 on sepsis-induced abnormal kidney function, the WT mice were preinjected with an S100A8/A9 specific inhibitor (Paq, 10 mg/kg) prior to CLP surgery. This dosage of Paq at 10 mg/kg was selected based on our recent studies (Wu et al., 2023; Zhang et al., 2023). After 48 h, the blood samples were collected to evaluate the markers for renal function (Figure 2A). Compared to the vehicle + sham group, CLP surgery significantly increased serum urea nitrogen (BUN) and creatinine (Cr) levels. Notably, Paq at 10 mg/kg markedly attenuated this effect (Figure 2B). Thus, reviewing relevant literature and setting a concentration gradient, we observed that the optimal drug concentration of Paq was 10 mg/kg. Moreover, we detected the level of serum S100A9, and found that serum S100A9 in mice was significantly increased after Paq treatment and CLP surgery (Figure 2C). Finally, we observed that Paq administration slightly improved the survival rate of CLP-treated mice at 48 h compared with CLP group in data (Figure 2D). In all, these data point that Paq can ameliorate sepsis-induced kidney dysfunction.

### 3.3 Administration of Paquinimod attenuates renal pathological changes and apoptosis

**FIGURE 3:**
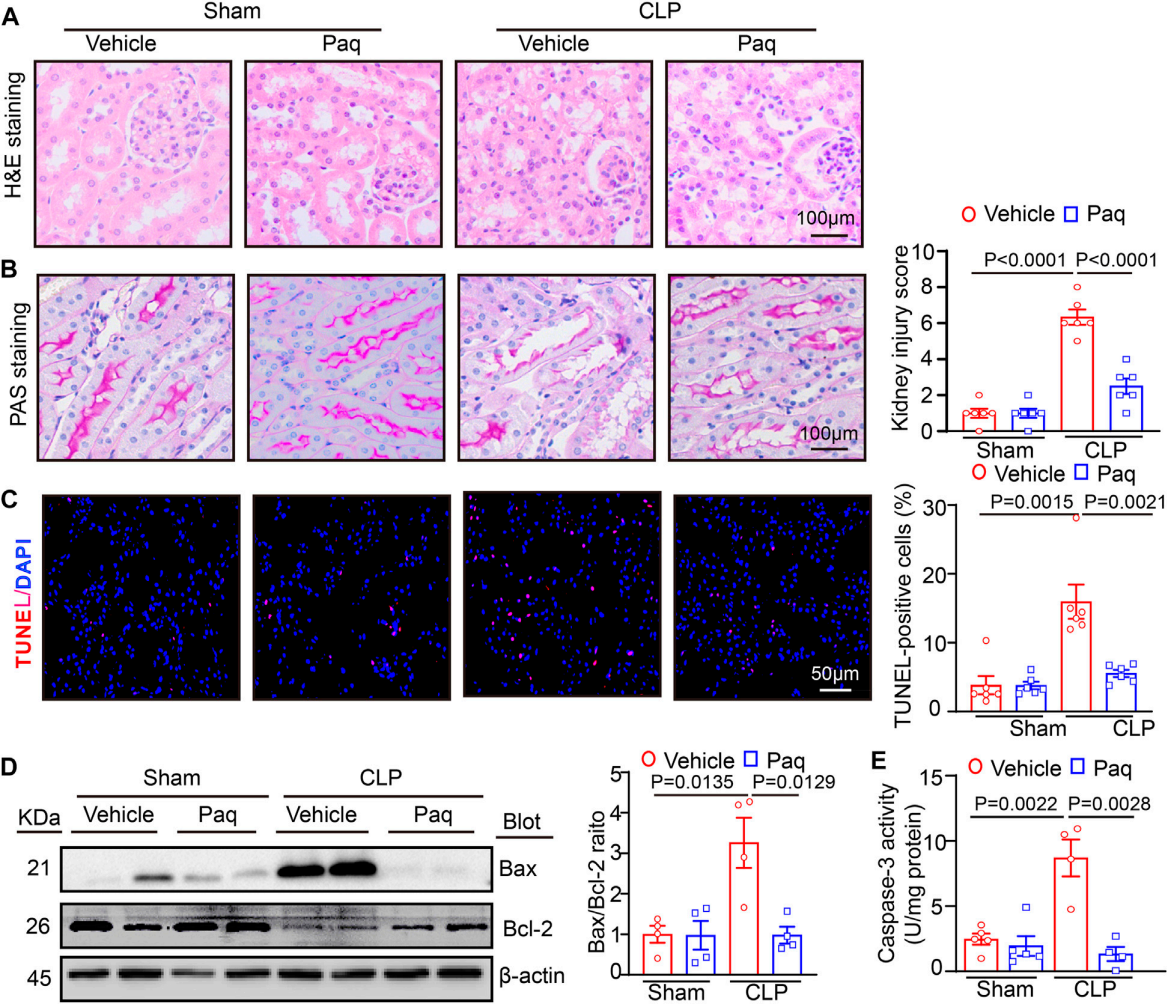
Blocking S100A9 with Paq suppresses CLP-induced pathological changes and cell apoptosis in the kidneys. The WT mice were pretreated with Paq (10 mg/kg) before CLP surgery and after 48 h. **(A)** Haematoxylin and eosin (H&E) staining of the kidney sections (left). Scale bars: 100 μm. **(B)** Periodic acid-Schiff (PAS) staining of the kidney sections (left) (Scale bars: 100 μm) and quantifying the tubular damage score based on PAS staining (right, *n* = 6 per group). **(C)** TUNEL (red) and DAPI (blue) staining of the kidney sections (left) and quantifying TUNEL-positive apoptotic cells (*n* = 6 per group). Scale bars: 50 μm. **(D)** Immunoblotting analysis of Bax and Bcl-2 protein levels in the kidneys (left) and quantifying the relative Bax/Bcl-2 ratio level (*n* = 4 per group). **(E)** ELISA analysis of caspase-3 activity in the kidney tissues (*n* = 4 or 5 per group).

Then, we tested whether the inhibition of S100A9 improves pathological changes in the kidney. H&E and PAS staining revealed that CLP surgery significantly augmented renal tubular injury scores, as reflected by increased swelling, vacuolation, necrosis, irregular brush border, and ectasia of the proximal tubular epithelial cells. However, these deleterious effects were markedly reduced in the kidneys of Paq-treated mice (Figures 3A, B). Moreover, we proved that inhibition of S100A9 could decrease the apoptosis of cells in kidney. TUNEL exhibited that the number of apoptotic cells, as indicated by TUNEL-positive in the kidneys of the Paq-treated group was lower than that in the vehicle-treated group after CLP surgery (Figure 3C). What’s more, we detected the content of the apoptotic related proteins (Bax and Bcl-2) and Caspase-3 activity with immunoblotting and ELISA kit, respectively. These data proved that Paq treatment substantially reduced the CLP-mediated increase of Bax/Bcl-2 ratio levels and Caspase-3 activity in the kidneys compared with vehicle treatment after CLP (Figures 3D, E). Altogether, these findings proved that the inhibition of S100A9 exerts a protective role in septic kidney injury.

### 3.4 Administration of Paquinimod inhibits renal macrophage infiltration and oxidative stress and increases cell proliferation in renal tissue

**FIGURE 4:**
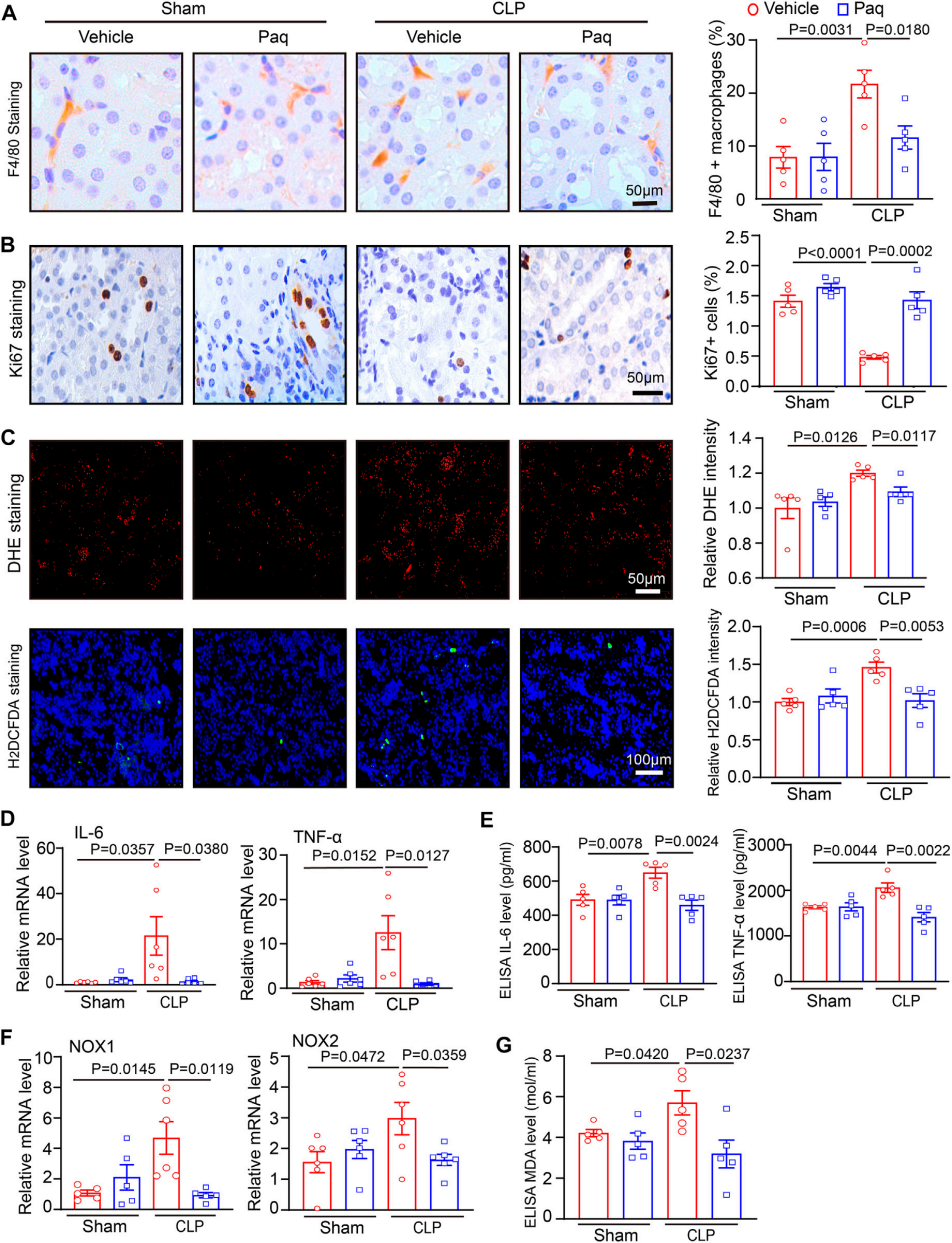
Blockage of S100A9 with Paq reduces CLP-induced macrophage infiltration and oxidative stress in the kidneys. WT mice were pretreated with Paq (10 mg/kg) and then applied to CLP surgery for 48 h **(A,B)** Immunohistochemical staining of kidney slices with anti-F4/80 antibody and anti-ki67 (left) and quantifying F4/80-positive macrophages (right, *n* = 5 per group) and ki67-positive cells (right, *n* = 5 per group). Scale bars: 50 μm. **(C)** DHE and H_2_DCFDA staining of kidney sections (left) and quantifying relative DHE and H_2_DCFDA fluorescence intensities (*n* = 5 per group). Scale bars: 50–100 μm. **(D,F)** qPCR analysis of IL-6, TNF-ɑ, NOX1, and NOX2 mRNA levels in the kidney tissues (*n* = 5 or 6 per group). **(E,G)** ELISA analysis of IL-6, TNF-α and MDA levels in the kidney tissues (*n* = 5 per group,IL-6,TNF-α).

Since S100A8/A9 can activate different receptors, such as TLR4, to promote leukocyte recruitment and secretion of proinflammatory cytokines, such as IL-6 and TNF-α, leading to kidney injury (Jung et al., 2019). Paq is a specific inhibitor that prevents the binding of S100A8/A9 to TLR4 (Jha et al., 2021; Wu et al., 2023). Therefore, we tested whether Paq can reduce renal inflammation. Immunohistochemical staining indicated that Paq administration for 48 h substantially inhibited CLP-induced infiltration of F4/80-positive macrophage and increased ki67-positive cell proliferation in the kidneys compared with vehicle treatment (Figures 4A, B). Moreover, oxidative stress is an important pathological occurrence in septic AKI (Carlsson et al., 2005). Next, we determined whether S100A8/A9 inhibition improved oxidative stress in the kidney. We performed DHE and H_2_DCFDA (a cell-permeable probe used to detect intracellular ROS level) staining in renal tissues. Our results showed that the relative fluorescence intensities of DHE and H_2_DCFDA staining were stronger in the kidneys of CLP-treated mice, and this enhancement was markedly blocked in the kidneys of Paq-treated mice (Figure 4C). Furthermore, qPCR results showed that the mRNA levels of proinflammatory cytokines (IL-6 and TNF-α) and NADPH oxidase isoforms (NOX1 and NOX2) were also lower in the kidneys of Paq-treated mice than those in the kidneys of CLP-treated mice (Figures 4D, F). Accordingly ELISA results proved that CLP-induced upregulation of IL-6, TNF-α and MDA levels were remarkable reversed in the kidneys of Paq-treated mice (Figures 4E, G). Thus, these data imply that the inhibition of S100A8/A9 binding to TLR4 reduces inflammation, oxidative stress, and increases cell proliferation in the kidneys during sepsis.

### 3.5 Administration of Paquinimod improves the balance of mitochondrial fission and fusion

**FIGURE 5:**
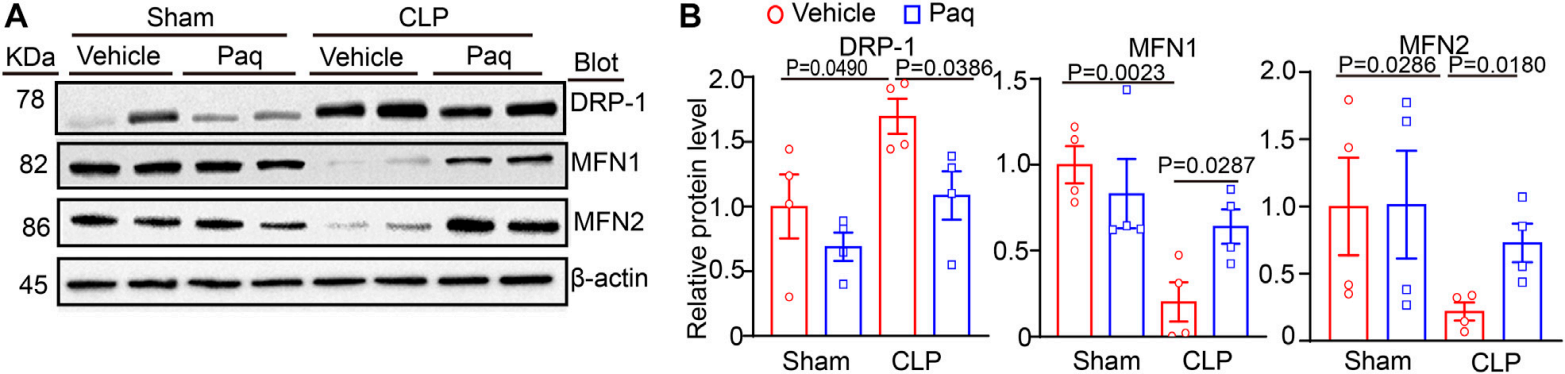
Inhibition of S100A9 with Paq reverses CLP-induced imbalance of mitochondrial fission and fusion in the kidneys. The WT mice were pretreated with Paq (10 mg/kg) before CLP surgery and after 48 h **(A,B)** Immunoblotting analysis of Drp1, Mfn1, and Mfn2 protein levels in the kidneys (left) and quantifying the relative protein level (*n* = 4 per group).

Studies have pointed that mitochondrial impairment is a key mechanism leading to cell apoptosis and oxidative stress, the hallmarks of AKI(21). To determine the possible molecular mechanism of S100A8/A9 in septic kidney injury, we examined the mitochondrial pro-fission protein Drp1, and pro-fusion proteins Mfn1 and Mfn2, which are important regulators of mitochondrial dynamic balance. Immunoblotting analysis indicated that after 48 h, CLP surgery resulted in a remarkable increase of Drp1 and reduction of Mfn1/2 in the kidneys compared with the sham operation. Still, this change was reversed in the Paq-treated kidneys (Figures 5A, B). This suggested that the inhibition of S100A9 may improve mitochondrial fission-fusion balance and dysfunction, leading to the inhibition of AKI after sepsis.

### 3.6 Paquinimod improves oxidative stress, apoptosis and the balance of mitochondrial fission and fusion in mouse renal tubular epithelial cells (mRTECs)

**FIGURE 6:**
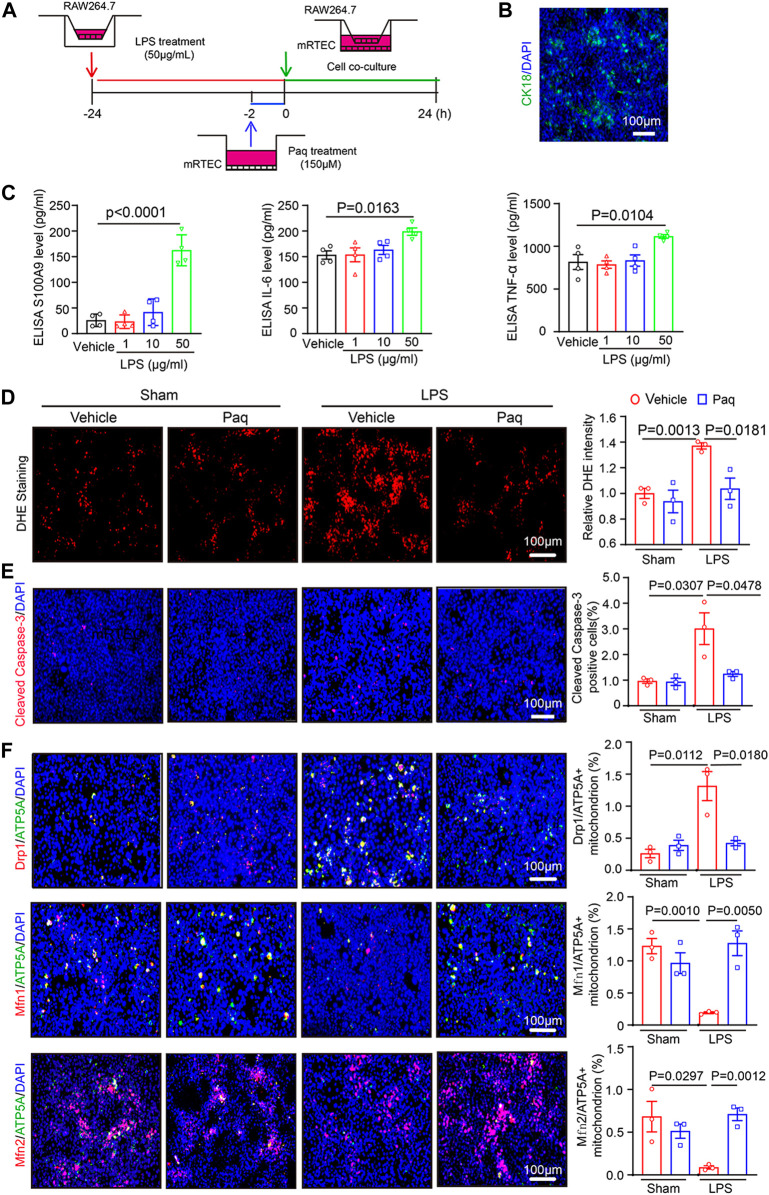
Blockage of S100A8/A9 with Paq reduces LPS-induced inflammation, oxidative stress, apoptosis, and mitochondrial fission and fusion-related proteins in the mRTEC cells. **(A)** RAW264.7 cells were pretreated with LPS (50 μg/mL) and then co-cultured with the mRTEC cells for 24 h, which were preinjected with Paq (150 μM) for 2 h. **(B)** Immunofluorescence staining of mRETC cells with CK18 antibody. **(C)** ELISA analysis of S100A9, IL-6 and TNF-α levels in the supernatants of cultured RAW264,7 cells (*n* = 4 per group). **(D)** DHE staining of mRTEC cells (left) and quantifying relative DHE fluorescence intensity (*n* = 3 per group). Scale bars: 100 μm. **(E)** Immunofluorescence staining of mRETC cells with cleaved caspase-3 antobody. (left) and quantifying apoptosis cells (*n* = 3 per group). Scale bars: 100 μm. **(F)** Immunofluorescence double staining of mRTEC cells with Drp1/ATP5A, Mfn1/ATP5A, Mfn2/ATP5A antibodies (left) and quantifying relative positive mitochondrion.

To further verify the role of Paq in septic nephropathy, we used cell co-culture to confirm relevant experimental results. We selected RAW264.7 macrophages and mRTEC cells for co-culture to simulate the mode of action of macrophages on renal tubular epithelial cells *in vivo*. Before co-culture, the RAW264.7 macrophages were pretreated with different dosages of LPS (1, 10 and 50 μg/mL) for 24 h and mRTEC cells were pretreated with Paq (150 μM) for 2 h (Figure 6A). ELISA assay indicated that LPS treatment at 50 μg/mL significantly increased levels of S100A9, IL-6, and TNF-α in the culture medium of RAW264.7 cells when compared with vehicle control (Figure 6C). Next, we co-cultured RAW 264.7 and mRTEC cells, and detected the oxidative stress and apoptosis level of mRTEC cells with DHE and cleaved caspase-3 staining, respectively. We found that the relative DHE fluorescence intensity and caspase-3-positive apoptosis were higher in the mRTEC cells of LPS-treated group, and this enhancement was remarkable blocked in the Paq-treated group (Figures 6D, E). Finally, we used double immunostaining to verify the mitochondrial fission and fusion. Drp1/ATP5A double staining show that Drp1-positive content in mitochondrion was remarkably increased in LPS-treated mRTECs, and this effect was markedly attenuated in Paq-treated mRTECs. Conversely. Mfn1/ATP5A and Mfn2/ATP5A staining show that Mfn1 and Mfn2 content in mitochondrion is downregulation in LPS-treated mRTECs, and this reduction was greatly reversed in Paq-treated cells (Figure 6F). Collectively, these results demonstrate that inhibition of S100A9 can ameliorate the imbalance of mitochondrial fission and fusion in mRTECs induced by LPS.

## 4 Discussion

**FIGURE 7:**
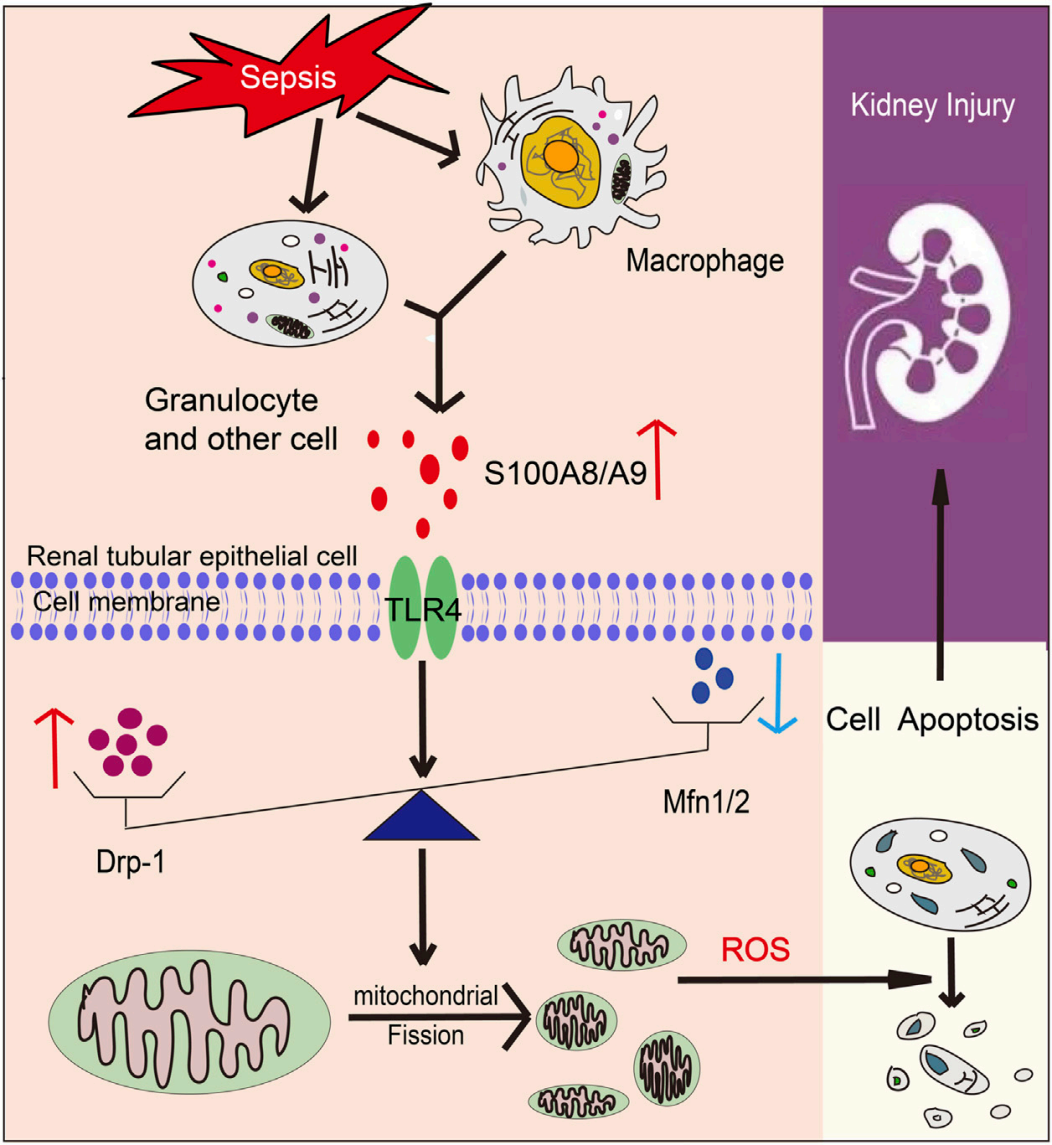
A working model for S100A8/A9 in regulating sepsis-induced acute kidney injury (AKI). Upon sepsis, S100A9 expression is upregulated and released by macrophages promoting activation of TLR4, which increases Drp1 level and reduces Mfn1/2 level that cause mitochondrial fission-fusion imbalance, excessive oxidative stress and inflammation, leading to epithelial apoptosis and AKI.

In this paper, we identified a novel role of S100A9 on macrophages in facilitating sepsis-induced AKI and abnormal kidney function. Our results proved that S100A9 expression was highly regulated upwards in the kidneys and macrophages of CLP-treated mice. Blockage of S100A9 with Paq substantially ameliorated CLP-induced abnormal renal function, apoptosis, inflammation, and oxidative stress in tubular epithelial cells likely associated with improving the balance of Drp1/Mfn1/2-mediated mitochondrial dynamics. The results are summarized in Figure 7. Therefore, this study demonstrates that S100A9 has an impact on sepsis-induced AKI, and emphasizes targeting S100A9 as a potential therapeutic option for this disease.

Kidney is may the most common organ which is affected during sepsis. More and more evidence has illuminated that AKI is the leading contributor to sepsis-related morbidity and mortality, and up to 50%–60% of patients with sepsis have AKI (Peerapornratana et al., 2019). In septic AKI, the kidney undergoes pathological changes, including infiltration of inflammatory cells, renal tubular dilation, vacuolisation, swelling and necrosis of epithelial cells, obstruction and distortion of renal tubules, and disappearance of the tubular brush border (Peerapornratana et al., 2019; Wei et al., 2019). In this study, these pathological changes were observed in both H&E and PAS staining, and were improved by use of S100A8/A9 inhibitor Paq. Several studies have demonstrated that proinflammatory cascades are key mediator of septic AKI. As an important inflammatory mediator, S100A8/A9 is highly expressed in stimulated and activated monocytes/macrophages and Ly6G-positive granulocytes in ischemia/reperfusion (I/R) kidney and obstructive nephropathy (Dessing et al., 2015; Tammaro et al., 2018; Wu et al., 2023; Zhang et al., 2023) and can be released during inflammation and cell necrosis (Carlsson et al., 2005; Wang et al., 2018). Here, our data further confirmed that the S100A9 was expressed in macrophages after CLP and LPS stimulation (Figure 1). Moreover, increased S100A8/A9 expression is associated with inflammation and cell death (Alaedini et al., 2008), and promotes proliferation, differentiation, and apoptosis of tumor cells, epithelial cells, monocyte, and smooth muscle cells (Jukic et al., 2021) Importantly, S100A8/A9 (as an important DAMPs) are involved in various diseases, such as I/R- and contrast-induced AKI, renal fibrosis, and glomerulonephritis in different animal models, via TLR4-or RAGE-mediated activation of multiple signals, and proinflammatory cytokines production (Pepper et al., 2015; Tan et al., 2017; Austermann et al., 2018; Du et al., 2022; Yao et al., 2022). However, there are few studies on the role of S100A8/A9 in septic AKI. In this study, we found that Paq treatment highly enhanced serum levels of S100A9 in mice (Figure 2C). This result may be due to the competitive binding of Paq to TLR4 receptors in tissues, which reduces the binding of S100A8/A9 to TLR4 receptors, finally leads to the increase of free S100A9. Moreover, we discovered for the first time that administration of S100A9 inhibitor Paq remarkable hinders the binding of S100A9 to TLR4. These results buttressed previous findings and confirmed that S100A9 exerts a protective role in septic AKI, as indicated by the reduction of animal mortality and improvement of renal dysfunction and pathological changes including cell apoptosis, oxidative stress, and inflammatory response in CLP-treated mice (Figures 2–4), confirming that S100A9 critically contributes to CLP-induced AKI and abnormal kidney function.

Recent advances in sepsis-related organ injury in different animal models have increased our knowledge of the pathogenesis of AKI. More and more evidences point that mitochondrial dysfunction may act as a pivotal cause to sepsis-induced organ failure, including renal injury and abnormal kidney function (Sun et al., 2019). Mitochondrial dynamic balance is a pivotal process in normal mitochondrial morphology and function. Several Key dynamin-related GTPases are involved in regulating mitochondrial fusion and fission balance, including Drp1, and optic atrophy1 (OPA1) (Kulek et al., 2020). Among these proteins, Drp1 forms a multimeric complex that wraps the mitochondria to induce mitochondrial fission, whereas Mfn1/2 and OPA1 can fuse the outer and inner mitochondrial membranes to drive mitochondrial fusion (Kulek et al., 2020; Monteith et al., 2022). In response to injuries such as sepsis, the balance of mitochondrial fission and fusion is disrupted, and mitochondria tend to divide [34]. Mitochondrial dynamics are characterized by aberrant cycle of fission/fusion, however, they were more inclined to fission. During AKI in sepsis, mitochondrial fission-associated oxidative stress, apoptosis and pyroptosis were elevated (Liu et al., 2019; Li et al., 2020). Inhibition of Drp1 with its inhibitor Mdivi-1 improve mitochondrial fission and limits the activation of NLRP3-mediated pyroptosis, confirming the role of Drp1 in septic AKI (Liu et al., 2020). SENP3-mediated Drp1 deSUMOylation aggravates LPS-mediated tubular epithelial cell apoptosis during AKI. In contrast, SENP3 knockdown markedly ameliorates LPS-induced effects (Wang et al., 2022). Studies have shown that intracellular accumulation of S100A9 is associated with impaired mitochondrial homeostasis (Monteith et al., 2022). Our results confirmed that S100A9 enhanced sepsis-mediated mitochondrial fission-fusion imbalance, ultimately resulting in excessive oxidative stress and epithelial cell apoptosis of kidney. Conversely, the inhibition of S100A9 can improve these deleterious effects by reducing Drp1 and increasing Mfn1/2 protein levels (Figures 3–5). Here, the protein levels of Drp1 and Mfn1/2 were measured using total proteins from the kidneys tissues at a lower cost with simplicity and ease. However, this approach does not exactly reflect the change of mitochondrial mitochondrial fission and fusion proteins. Thus, we added the mitochondrial markers ATP5A and the mitochondrial fission proteins Drp1, mitochondrial fusion proteins MFN1, MFN2 immunofluorescence double staining to further prove the situation of mitochondrial division and fusion in cell experiment (Figure 6).

Currently, because the underlying pathophysiological processes of AKI in sepsis are not fully understood, this inevitably hinders the effective therapeutic interventions developing. The use of diuretics has not been demonstrated to inhibit or attenuate septic AKI (Peerapornratana et al., 2019). Thus, the routine administration of diuretics for the prevention or treatment of AKI cannot be recommended. Numerous drugs for AKI have been explored. To date, several molecules such as AC607, AB103, ABT-719AB103 (AtoxBio), ciclosporin A, and levosimendan have been examined to prevent or treat AKI in septic animal models and clinical trials. These agents can improve inflammatory response, oxidative stress, and mitochondrial impairment in the kidney during sepsis and other high-stress settings (Gallagher et al., 2017). Moreover, systemic use of alkaline phosphatase can protect against sepsis-induced AKI. This effect is associated with the direct dephosphorylation of endotoxin, which leads to improves survival rates and inhibits of inflammation and organ damage (Pickkers et al., 2018; Singh and Lin, 2021). Moreover, some caspase and interleukin inhibitors have not translated into human investigations of septic AKI (Takeyoshi et al., 2005; Yang et al., 2021). In this study, we used a variety of methods to verify the important role of S100A8/A9 in septic nephropathy *in vivo* and *in vitro*. We used the S100A8/A9 specific inhibitor Paquinimod to demonstrate for the first time that selective inhibition of S100A8/A9 reduces inflammatory responses, oxidative stress, and tubular epithelial apoptosis in septic nephropathy. Immunofluorescence double staining experiment *in vitro* confirmed that S100A8/A9 can increase the mitotic protein and decrease the fusion protein. The mechanism of S100A8/A9 was discussed for the first time. Our paper suggest that S100A8/A9 may be an important new point for the treatment of septic AKI, and provides a theoretical basis for the subsequent drugs development and clinical treatment of septic nephropathy.

This study has several limitations. First, the effect of S100A8/A9 on septic AKI needs to be clarified in S100A9 knockout mice and other animal models of sepsis. Secondly, the precise mechanism for S100A8/A9 to regulate mitochondrial dynamic balance is required to elucidated in further studies.

## 5 Conclusion

Our study demonstrates the damaging role of S100A8/A9 in macrophages in sepsis-induced AKI in mice. Inhibition of S100A9 ameliorated renal inflammation, oxidative stress, and epithelial cell apoptosis associated with mitochondrial Drp1/Mfn1/2 imbalance. Our study also emphasizes that S100A8/A9 may be a potential treated aspect for septic AKI.

## Data Availability

The raw data supporting the conclusion of this article will be made available by the authors, without undue reservation.
